# Association of circulating MR-proADM with all-cause and cardiovascular mortality in the general population: Results from the KORA F4 cohort study

**DOI:** 10.1371/journal.pone.0262330

**Published:** 2022-01-06

**Authors:** Christina Gar, Barbara Thorand, Christian Herder, Chaterina Sujana, Margit Heier, Christa Meisinger, Annette Peters, Wolfgang Koenig, Wolfgang Rathmann, Michael Roden, Michael Stumvoll, Haifa Maalmi, Thomas Meitinger, Holger Then, Jochen Seissler, Cornelia Then

**Affiliations:** 1 Department of Medicine IV, University Hospital, LMU Munich, Germany; 2 Clinical Cooperation Group Diabetes, Ludwig-Maximilians-Universität München and Helmholtz Zentrum München, Munich, Germany; 3 German Center for Diabetes Research (DZD), München-Neuherberg, Germany; 4 Institute of Epidemiology, Helmholtz Zentrum München, German Research Center for Environmental Health (GmbH), Neuherberg, Germany; 5 Institute of Clinical Diabetology, German Diabetes Center, Leibniz Center for Diabetes Research at Heinrich Heine University Düsseldorf, Düsseldorf, Germany; 6 Division of Endocrinology and Diabetology, Medical Faculty, Heinrich Heine University Düsseldorf, Düsseldorf, Germany; 7 Institute for Medical Information Processing, Biometry, and Epidemiology (IBE), Ludwig-Maximilians-Universität, Munich, Germany; 8 KORA Study Centre, University Hospital Augsburg, Augsburg, Germany; 9 Independent Research Group Clinical Epidemiology, Helmholtz Zentrum München, German Research Center for Environmental Health (GmbH), Neuherberg, Germany; 10 Chair of Epidemiology at University Hospital Augsburg, Augsburg, Germany; 11 DZHK (German Centre for Cardiovascular Research), Partner Site Munich Heart Alliance, Munich, Germany; 12 Institute of Epidemiology and Medical Biometry, University of Ulm, Ulm, Germany; 13 Deutsches Herzzentrum München, Technische Universität München, Munich, Germany; 14 German Diabetes Center, Leibniz Institute at Heinrich Heine University Düsseldorf, Institute of Biometrics and Epidemiology, Düsseldorf, Germany; 15 Department of Medicine, University of Leipzig, Leipzig, Germany; 16 Institute of Human Genetics, Technische Universität München, Munich, Germany; 17 Freie Waldorfschule Augsburg, Augsburg, Germany; Kasturba Medical College Mangalore, INDIA

## Abstract

**Background and aim:**

Despite its vasodilatory effect, adrenomedullin and its surrogate mid-regional pro-adrenomedullin (MR-proADM) have been found to be positively associated with all-cause and cardiovascular mortality. However, the underlying mechanisms thereof remain unclear and the associations were mostly shown in geriatric cohorts or in patients with chronic diseases. Therefore, we aimed to investigate the possible involvement of abdominal obesity, selected adipokines, and biomarkers of subclinical inflammation in the association of MR-proADM with mortality in a population based study cohort.

**Methods:**

Prospective analysis of the KORA F4 study; median follow-up 9.1 (8.8–9.4) years. Complete data on MR-proADM and mortality was available for 1551 participants, aged 56.9±12.9 years (mean±SD). Correlation and regression analyses of MR-proADM with overall (BMI) and abdominal obesity (waist circumference), selected adipokines and biomarkers of subclinical inflammation. Cox proportional hazard models on the association of MR-proADM with all-cause and cardiovascular mortality with adjustment for cardiovascular risk factors and selected biomarkers in study subgroups (n = 603–1551).

**Results:**

MR-proADM associated with all-cause (HR (95%CI): 2.37 (1.72–3.26) and 2.31 (1.67–3.20)) and cardiovascular mortality (4.28 (2.19–8.39) and 4.44 (2.25–8.76)) after adjustment for traditional cardiovascular risk factors including BMI or waist circumference, respectively. MR-proADM was further associated with four out of seven examined adipokines (leptin, retinol-binding protein-4, chemerin, and adiponectin) and with five out of eleven examined biomarkers of subclinical inflammation (high-sensitivity C-reactive protein, interleukin-6, myeloperoxidase, interleukin-22, and interleukin-1 receptor antagonist) after multivariable adjustment and correction for multiple testing. However, only IL-6 substantially attenuated the association of MR-proADM with all-cause mortality.

**Conclusions:**

We found an association of MR-proADM with (abdominal) obesity, selected adipokines, and biomarkers of subclinical inflammation. However, the association of MR-proADM with mortality was independent of these parameters. Future studies should investigate the role of IL-6 and further characteristics of subclinical inflammation in the association between MR-proADM and all-cause mortality.

## Introduction

Adrenomedullin (AMD) is a 52 amino acid peptide synthesized by a variety of tissues, including adrenal medulla, vascular endothelial cells, vascular smooth muscle cells and adipose tissue [1–3], and exhibits vasodilatative properties [4]. In addition to its hypotensive effects, biological actions of AMD include antioxidative and antiapoptotic but also metabolic effects, such as inhibition of insulin secretion in beta cells [4]. Increased AMD levels and its surrogate marker mid-regional pro-adrenomedullin (MR-proADM) are correlated with congestive heart failure, hypertension and incident cardiovascular events [4]. During recent years, ADM and MR-proADM have been shown to be associated with cardiovascular and all-cause mortality [4–6]. Mostly, these associations have been shown in geriatric cohorts or in patients with chronic diseases [7–12]. The underlying mechanisms that link (MR-pro)ADM to mortality remain to be determined [7–11].

Because the measurement of ADM itself is challenging due to its short half-life, MR-proADM as a surrogate for ADM levels has become increasingly prominent. MR-proADM represents a more stable peptide with a longer half-life and is co-secreted together with ADM from pre-pro-adrenomedullin, resulting in a direct association between plasma levels of ADM and MR-proADM [12].

Besides mortality, MR-proADM levels are linked to obesity [13–16] and the metabolic syndrome [17]. Given that obesity is a known risk factor for mortality [18], the association between MR-proADM and mortality might be mediated by their common association to body fat and other cardiovascular risk factors and thus be a secondary finding. One study, however, found that after adjustment for BMI, MR-proADM remained significantly associated with all-cause mortality, and thus, MR-proADM might affect mortality via mechanisms other than simple body fat mass [19]. In this context, abdominal fat mass was the only anthropometric index to be independently linked to MR-proADM levels [16]. Further, abdominal fat, as opposed to overall body fat, might be more relevant for an increased mortality rate in the context of the metabolic syndrome [2, 4, 13, 14, 20–22]. The BMI-independent association between MR-proADM and mortality might therefore be explained by varying levels of abdominal fat at comparable BMI levels. Further, an altered secretion profile of adipokines and markers of subclinical inflammation may indicate metabolic alterations in the adipose tissue [23–25]. Subclinical inflammation [26] as well as alterations in adipokine levels, e.g. adiponectin [27], were found to be associated with an elevated mortality risk. In this respect, ADM was suggested to exert both proinflammatory and immunomodulatory effects [4, 28–30]. Consequently, immunomodulatory adipokines and biomarkers of subclinical inflammation may also confound the relationship between MR-proADM and mortality.

Therefore, the present study aimed to investigate the association of MR-proADM with all-cause and cardiovascular mortality in the KORA (Cooperative Health Research in the Region of Augsburg) F4 cohort and to further examine whether the association between MR-proADM and mortality is independent of abdominal fat, adipokines, and biomarkers of subclinical inflammation.

## Materials and methods

### Study cohort

The KORA F4 (2006–2008) study is a follow-up examination of the population-based KORA S4 study (1999–2001). Recruitment and eligibility criteria for the KORA studies, study design, standardized sampling methods and data collection (medical history, medication, anthropometric and blood pressure measurements) have been described elsewhere [31, 32]. The outcomes all-cause and cardiovascular mortality (ICD-9 codes 390–459 and 798) were ascertained by regularly checking the status of the participants through the population registries until 2016. Death certificates were obtained from the local health authorities. Prevalent myocardial infarction and stroke were self-reported diagnoses. The median (1st; 3rd quartile) follow-up time was 9.1 (8.8; 9.4) years.

All study participants gave written informed consent. The study was approved by the Ethics Committees of the Bavarian Medical Association in adherence to the declaration of Helsinki.

MR-proADM was measured in the first 1596 participants of the KORA F4 study (out of a total of 3080 participants). All variables necessary for the analysis of the association of MR-proADM with mortality were available in 1551 participants. Measurements of leptin and retinol-binding protein-4 (RBP-4) were available in 1549 participants; chemerin in 1055 participants; wingless-type MMTV integration site family, member 5A (Wnt-5a), secreted frizzled-related protein-5 (SFRP-5), vaspin, progranulin, omentin, and adiponectin were available in 606 participants aged ≥ 62 years; interleukin-6 (IL-6), tumor necrosis factor-α (TNF-α), interleukin-18 (IL-18), soluble intercellular adhesion molecule-1 (sICAM-1), myeloperoxidase (MPO), superoxide dismutase-3 (SOD-3), interleukin-22 (IL-22) and interleukin-1 receptor antagonist (IL-1RA) were available in 603 participants aged ≥ 62 years.

Overall obesity was defined as a BMI ≥ 30 kg/m^2^. Abdominal obesity (increased waist circumference) was defined according to the International Diabetes Federation definition (waist circumference ≥ 94 cm in men and ≥ 80 cm in women) [33].

Criteria for diabetes mellitus were a validated medical diagnosis or current self-reported use of glucose-lowering agents. Participants without clinically diagnosed diabetes underwent a standard 75 g oral glucose tolerance test. Newly diagnosed diabetes was defined according to the World Health Organization diagnostic criteria (≥ 7.0 mmol/l fasting and/or ≥ 11.1 mmol/l 2-h glucose) [34]. Participants with a diabetes type other than type 2 or unknown glucose tolerance status (n = 25) were excluded from the analyses.

Arterial hypertension was defined as a systolic blood pressure ≥ 140 mmHg and/or a diastolic blood pressure ≥ 90 mmHg, and/or intake of anti-hypertensive medication, given that the participants were aware of being hypertensive.

Leisure-time physical activity was assessed with two separate questions concerning leisure time sports activity in winter and in summer (cycling included). Possible answers were (i) > 2 hours, (ii) 1–2 hours, (iii) < 1 hour and (iv) none per week. Participants who had a total score < 5, obtained by summing the numbers (i)-(iv) from the winter and summer questions, were classified as ‘physically active’.

### Biochemical measurements

Blood samples were collected after an overnight fast of at least eight hours and were kept at room temperature until centrifugation. Plasma was separated immediately, serum after 30 min. Plasma and serum samples were assayed immediately or stored at -80°C. Plasma MR-proADM was measured by sandwich fluoro-immunoassay (BRAHMS, Hennigsdorf, Berlin, Germany) on an automated BRAHMS KRYPTOR system as previously described [35]. Blood serum glucose levels were assessed using the hexokinase method (GLU Flex; Dade Behring, Marburg, Germany). High- and low-density lipoprotein (HDL and LDL) cholesterol were measured with enzymatic methods (CHOD-PAP; Dade Behring). Triglycerides were measured by an enzymatic color test (GPO-PAP method, TGL Flex; Dade Behring). Serum creatinine was determined with a modified Jaffe test (Krea Flex; Dade Behring). Insulin was measured by an electrochemiluminescence immunoassay on a Cobas e602 instrument (Roche Diagnostics GmbH, Mannheim, Germany). HbA1c was measured in hemolyzed whole blood using the cation-exchange high performance liquid chromatographic, photometric VARIANT II TURBO HbA1c Kit—2.0 assay on a VARIANT II TURBO Hemoglobin Testing System (Bio-Rad Laboratories Inc., Hercules, USA).

High-sensitivity C-reactive protein (hsCRP) was determined in plasma with a high-sensitivity latex-enhanced nephelometric assay on a BN II analyzer (Siemens, Erlangen, Germany). Serum levels of IL-6 and TNF-α were measured with Quantikine HS ELISA kits, IL-22, IL-1RA, sICAM-1, and MPO with Quantikine ELISA kits (R&D Systems, Wiesbaden, Germany) [5, 6, 36]. Plasma levels of IL-18 were determined using ELISA kits from MBL (Nagoya, Japan). Serum SOD-3 concentrations were measured with an ELISA from Cloud-Clone Corp. (Houston, TX, USA) [7]. Intra-assay coefficient of variation (CV) for hsCRP, IL-1RA, IL-22, sICAM-1, IL-6, TNF-α, IL-18, MPO, and SOD-3 were 2.7, 2.8, 5.5, 3.5, 7.2, 6.3, 7.6, 3.2, and 7.1%, respectively. Inter-assay CVs were 6.3, 7.0, 9.3, 6.4, 11.8, 14.4, 9.4, 5.6, and 7.1%, respectively. For IL-22, 332 (31%) of the sera yielded values below the limit of detection (LOD; 3.9 pg/ml). Values below LOD were assumed to be evenly distributed between 0 and LOD and were assigned a value of 0.5 × LOD. Chemerin serum concentrations were determined using a commercially available ELISA kit (Human Chemerin ELISA, Biovendor, Heidelberg, Germany) with a sensitivity of 0.1 ng/ml, an intra-assay CV of 6%, and an inter-assay CV of 7.6% [37]. Plasma concentrations of RBP-4 were measured by immunonephelometry using a BN II analyzer with an intra- and inter-assay CV of <10%. Leptin was measured by ELISA (Mercodia, Uppsala, Sweden); mean intra- and inter-assay CVs were <10% [38]. Serum levels of omentin-1 and adiponectin were measured using the Human Omentin-1 ELISA (BioVendor, Brno, Czech Republic) [39] and the Human Total Adiponectin/Acrp30 Quantikine ELISA Kit (R&D Systems) with intra-assay CVs of 2.0% and 4.0% respectively, and inter-assay CVs of 3.8 and 8.0%, respectively. Serum vaspin concentrations were measured using a commercial enzyme-linked immunosorbent assay kit (AdipoGen, Seoul, Korea) according to the manufacturer’s protocol [37]. The assay has a sensitivity of 12 pg/ml and the intra- and inter-assay CVs were 1.3–3.8% and 3.3–9.1%, respectively. Progranulin serum concentrations were determined using a commercially available ELISA (Progranulin human ELISA Kit AdipoGen, AdipoGen Inc., Korea) [37]. SFRP5 and Wnt-5a levels were measured using the Enzyme-linked Immunosorbent Assay Kit for Secreted Frizzled Related protein 5 (SFRP5) and Wnt-5a from Cloud-Clone (Houston, TX; previously USCN, Wuhan, China) [40]. All sera yielded levels above the LOD. Mean intra- and inter-assay CVs were 6.4 and 18.6%, respectively.

Glomerular filtration rate (eGFR) was calculated according to the Chronic Kidney Disease Epidemiology Collaboration (CKD-EPI) equation (2009) [41].

### Statistical analysis

Characteristics of the study participants were compared between participants with BMI < 30 kg/m^2^ (non-obese) and BMI ≥ 30 kg/m^2^ (obese), using t-tests in case of approximately normally distributed variables. Mann-Whitney U-tests were performed for variables with skewed distributions. Binomial proportions were compared with Chi-square tests. The associations of MR-proADM with all-cause and cardiovascular mortality were examined in Cox proportional hazard models. The cross-sectional associations of MR-proADM with categorical cardiovascular risk factors were assessed with logistic regression models, whereas the associations of MR-proADM with continuous outcomes were assessed using linear regression models. Continuous variables were transformed to a Gaussian distribution by the probability integral transformation followed by an inverse transform sampling and were used in calculations per one standard deviation. The associations of MR-proADM with the respective outcomes were examined in models without (crude model) and with adjustments for covariates:

Model 1: adjusted for sex and age (continuous);
Model 2: adjusted for, sex, age, and BMI (continuous);
Model 3: adjusted for sex, age, and *waist circumference* (continuous);
Model 4: adjusted for sex, age, BMI, diabetes, arterial hypertension, eGFR (continuous), HDL cholesterol (continuous), physical activity (active/inactive), current smoking and former smoking;
Model 5: adjusted for sex, age, *waist circumference*, diabetes, arterial hypertension, eGFR (continuous), HDL cholesterol (continuous), physical activity (active/inactive), current smoking and former smoking.

For further analyses, models adjusted for single parameters and adjustments according to model 4 plus single adipokines and biomarkers of subclinical inflammation were calculated.

LDL cholesterol was not found to be associated with MR-proADM after adjustment for sex, age, and BMI and was therefore not included in the models. Participants with missing data were excluded from the resptive adjusted and crude analyses. The level of statistical significance was set at 5% (two-sided). The Bonferroni method was used to correct for multiple testing where appropriate. The calculations were performed using the statistical environment R, version 3.6.0. Figures were created using Tableau 2020.4 (Tableau Software, Seattle, WA, USA).

## Results

### Clinical characteristics of the study participants

Table 1 shows the clinical characteristics of the study sample. MR-proADM was higher in obese (BMI ≥ 30 kg/m^2^) compared to non-obese (BMI < 30 kg/m^2^) participants. Besides the characteristics of the metabolic syndrome, obese participants also presented with a lower eGFR and were less physically active, but more likely to have quit smoking. Sex and a history of myocardial infarction or stroke did not differ between the groups. Several of the selected biomarkers (vaspin, progranulin, omentin, adiponectin, Wnt-5a, SFRP-5, sICAM-1, MPO, SOD-3, TNF-α, IL-6, IL-18, IL-22, and IL-1RA) were available only in participants aged ≥ 62 years.

**Table 1:** Characteristics of the KOFA F4 study participants, overall and stratified by BMI.

Characteristic	Total study cohort	Non-obese	Obese	p value ^#^
**All participants**	**N = 1551**	**N = 1160**	**N = 391**	-
Male sex, n (%)	762 (49)	574 (49)	189 (48)	0.674 ^e^
Age (years)	56.9 ± 12.9	55.9 ± 13.0	59.9 ± 12.0	< 0.001 ^c^
BMI (kg/m^2^)	27.4 ± 4.6	25.4 ± 2.7	33.6 ± 3.4	< 0.001 ^c^
Waist circumference (cm)	93.4 ± 13.6	88 ± 10.7	107.7 ± 10.9	< 0.001 ^c^
LDL cholesterol (mmol/l)	3.47 (2.88; 4.03)	3.44 (2.85; 4.01)	3.58 (2.95; 4.13)	0.035 ^d^
HDL cholesterol (mmol/l)	1.40 (1.16; 1.68)	1.47 (1.21; 1.73)	1.27 (1.11; 1.50)	< 0.001 ^d^
Type 2 diabetes, n (%)	181 (12)	82 (7)	99 (25)	< 0.001 ^e^
HbA1c (%)	5.4 (5.2; 5.7)	5.4 (5.1; 5.6)	5.6 (5.3; 5.9)	< 0.001 ^d^
HbA1c (mmol/mol)	35.5 (33.3; 38.8)	34.4 (32.2; 37.7)	37.7 (34.4; 41.0)	< 0.001 ^d^
Arterial hypertension, n (%) ^b^	617 (40)	377 (32)	241 (61)	< 0.001 ^e^
Systolic blood pressure (mmHg)	120.5 (109; 133)	119.0 (107.5; 131.5)	125.5 (115.0; 137.3)	< 0.001 ^d^
Diastolic blood pressure (mmHg)	74 (68.5; 81.5)	73.5 (68.0; 80.5)	76.0 (70.5; 84.0)	< 0.001 ^d^
eGFR (ml/min/1.73 m^2^)	88.4 (77.0; 98.9)	90.2 (78.4; 100.3)	82.7 (72.0; 95.0)	< 0.001 ^d^
Physically inactive, n (%)	657 (42)	452 (39)	205 (52)	< 0.001 ^e^
Current smoking, n (%)	273 (18)	225 (19)	48 (12)	0.002 ^e^
Former smoking, n (%)	639 (41)	452 (39)	187 (48)	0.003 ^e^
Previous myocardial infarction n (%)	52 (3)	35 (3)	17 (4)	0.274 ^e^
Previous stroke n (%)	34 (2)	11 (3)	23 (2)	0.445 ^e^
hsCRP (mg/l)	1.11 (0.55–2.43)	0.91 (0.47–1.88)	2.01 (1.06–4.22)	< 0.001 ^d^
MR-proADM (nmol/l)	0.51 (0.42; 0.61)	0.48 (0.41; 0.57)	0.59 (0.51; 0.71)	< 0.001 ^d^
	**N = 1549**	**N = 1158**	**N = 391**	
Leptin (ng/ml)	11.8 (5.4; 23.9)	8.7 (4.4; 16.7)	28.5 (15.7; 48.9)	< 0.001 ^d^
RBP-4 (g/l)	0.042 (0.035; 0.049)	0.042 (0.035; 0.049)	0.043 (0.037; 0.051)	< 0.001 ^d^
	**N = 1055**	**N = 757**	**N = 298**	
Chemerin (ng/ml)	172.2 (141.2; 205.7)	166.5 (135.8; 197.7)	194.9 (159.5; 230.2)	< 0.001 ^d^
Progranulin (ng/ml)	126.4 (99.4; 170.8)	125.6 (99.2; 164.9)	128.8 (99.8; 185.9)	0.130 ^d^
Vaspin (ng/ml)	0.61 (0.34; 1.14)	0.57 (0.32; 1.08)	0.72 (0.39; 1.31)	0.002 ^d^
**Participants aged ≥ 62 years**	**N = 606**	**N = 422**	**N = 184**	
Age (years)	70.1 ± 5.5	70.0 ± 5.6	70.3 ± 5.2	0.391 ^c^
BMI (kg/m2)	28.4 ± 4.2	26.2 ± 2.4	33.4 ± 3.1	< 0.001 ^c^
Waist circumference (cm)	97.7 ± 12.0	92.9 ± 9.6	108.4 ± 9.9	< 0.001 ^c^
MR-proADM (nmol/l)	0.60 (0.52; 0.71)	0.58 (0.50; 0.67)	0.67 (0.57; 0.79)	< 0.001 ^d^
Adiponectin (μg/ml)	10.0 (6.6; 15.3)	10.5 (7.1; 15.7)	9.1 (6.1; 14.0)	0.012 ^d^
Omentin (ng/ml)	480.7 (398.3; 580.1)	491.0 (413.3; 589.5)	454.8 (373.1; 556.5)	0.001 ^d^
SFRP-5 (ng/ml)	55.9 (42.5; 70.4)	57.9 (44.1; 73.0)	50.2 (37.1; 66.4)	< 0.001 ^d^
Wnt-5a (ng/ml)	0.038 (0.021; 0.077)	0.038 (0.021; 0.080)	0.037 (0.019; 0.066)	0.433 ^d^
	**N = 603**	**N = 420**	**N = 183**	
IL-6 (pg/ml)	1.52 (1.04; 2.28)	1.41 (0.93; 2.03)	1.82 (1.42; 2.61)	< 0.001 ^d^
TNF-α (pg/ml)	2.02 (1.46; 2.99)	1.94 (1.41; 2.82)	2.27 (1.62; 3.23)	0.003 ^d^
IL-18 (pg/ml)	319.0 (254.0; 416.5)	315.0 (251.0; 417.0)	325.0 (263.0; 415.5)	0.254 ^d^
sICAM-1 (ng/ml)	232.2 (199.8; 260.9)	225.6 (195.4; 256.9)	239.0 (206.9; 272.7)	0.002 ^d^
MPO (ng/ml)	154.9 (96.55; 223.9)	154.0 (93.0; 224.5)	161.1 (107.7; 218.4)	0.487 ^d^
SOD-3 (ng/ml)	127.1 (112.2; 143.0)	125.9 (111.8; 141.0)	130.6 (114.8; 147.4)	0.035 ^d^
IL-22 (pg/ml)	6.24 (1.95; 13.27)	5.99 (1.95; 13.33)	7.06 (1.95; 12.97)	0.182 ^d^
IL-1RA (pg/ml)	303.9 (235.2; 409.0)	283.1 (222.3; 365.8)	370.65 (300.9; 455.4)	< 0.001 ^d^

Non-obese: BMI < 30 kg/m^2^, obese: BMI ≥ 30 kg/m^2^.

^a^ data are given as mean ± SD, median (first quartile; third quartile), or number of participants (proportion in %);

^b^ defined as systolic blood pressure ≥ 140 mmHg and/or diastolic blood pressure ≥ 90 mmHg and/or use of antihypertensive medication, given that the participants were aware of being hypertensive;

^c^ t-test;

^d^ Mann-Whitney U-test;

^e^ Chi-square test.

### Association of MR-proADM with cardiovascular risk factors

First, we analyzed the association between MR-proADM and selected cardiovascular risk factors (Table 2). Except for sex, all selected cardiovascular risk factors (age, BMI, waist circumference, arterial hypertension, type 2 diabetes, LDL and HDL cholesterol, former and current smoking, and physical activity) significantly associated with MR-proADM in the crude model. All of these risk factors but LDL cholesterol remained significantly associated after adjustment for sex, age, and BMI.

**Table 2:** Association of MR-proADM (as dependent variable) with cardiovascular risk factors (as independent variables).

	β-coefficient ± SE	p-value	β-coefficient ± SE	p-value
	crude		Model 2^a^	
Male sex	0.01 ± 0.05	0.874	-	-
Age (years)	0.63 ± 0.02	< 0.001	-	-
BMI (kg/m^2^)	0.46 ± 0.03	< 0.001	-	-
Waist circumference (cm)	0.46 ± 0.03	< 0.001	-	-
Type 2 diabetes (yes/no)	0.97 ± 0.08	< 0.001	0.25 ± 0.08	0.003
Arterial hypertension (yes/no)	0.86 ± 0.05	< 0.001	0.19 ± 0.05	< 0.001
LDL cholesterol (mmol/l)	0.07 ± 0.03	0.008	-0.02 ± 0.02	0.203
HDL cholesterol (mmol/l)	-0.14 ± 0.03	< 0.001	-0.09 ± 0.02	0.003
eGFR (ml/min/1.73 m^2^)	-0.63 ± 0.02	< 0.001	-0.39 ± 0.03	< 0.001
Current smoking (yes/no) ^a^	0.12 ± 0.07	0.222	0.28 ± 0.05	< 0.001
Former smoking (yes/no) ^a^	0.17 ± 0.06	0.003	0.14 ± 0.04	< 0.001
Physical activity (yes/no)	-0.34 ± 0.05	< 0.001	-0.15 ± 0.04	< 0.001

^a^ Model 2: adjusted for sex, age, and BMI.

### Association of MR-proADM with all-cause and cardiovascular mortality

MR-proADM was associated with all-cause mortality. The association was affected by age, but hardly attenuated by the other cardiovascular risk factors. In all models, MR-proADM remained independently associated with all-cause mortality (p < 0.001; Table 3).

**Table 3:** Hazard ratios (95% confidence interval) of the association between MR-proADM and all-cause mortality (per 1-standard deviation).

	HR (95% CI)	p-value
**Adjustment for single risk factors**		
Crude	3.22 (2.70–3.82)	< 0.001
adjusted for sex	3.25 (2.74–3.85)	< 0.001
adjusted for age	2.01 (1.60–2.52)	< 0.001
adjusted for BMI	3.53 (2.92–4.28)	< 0.001
adjusted for waist circumference	3.13 (2.58–3.80)	< 0.001
adjusted for diabetes	3.08 (2.57–3.69)	< 0.001
adjusted for arterial hypertension	2.97 (2.46–3.59)	< 0.001
adjusted for HDL	3.26 (2.74–3.90)	< 0.001
adjusted for eGFR	2.87 (2.22–3.72)	< 0.001
adjusted for smoking	3.31 (2.77–3.96)	< 0.001
adjusted for physical activity	3.14 (2.63–3.76)	< 0.001
**Multivariable adjusted models**		
**Model 1**: adjusted for sex and age	2.08 (1.67–2.60)	< 0.001
**Model 2**: adjusted for sex, age, BMI	2.18 (1.70–2.78)	< 0.001
**Model 3**: adjusted for sex, age, waist circumference	2.09 (1.63–2.69)	< 0.001
**Model 4**: adjusted for sex, age, BMI, eGFR, arterial hypertension, diabetes, HDL, smoking, physical activity	2.37 (1.72–3.26)	< 0.001
**Model 5**: adjusted for sex, age, waist circumference, eGFR, arterial hypertension, diabetes, HDL, smoking, physical activity	2.31 (1.67–3.20)	< 0.001

n = 1551 (number of events n = 138).

**Fig 1:**
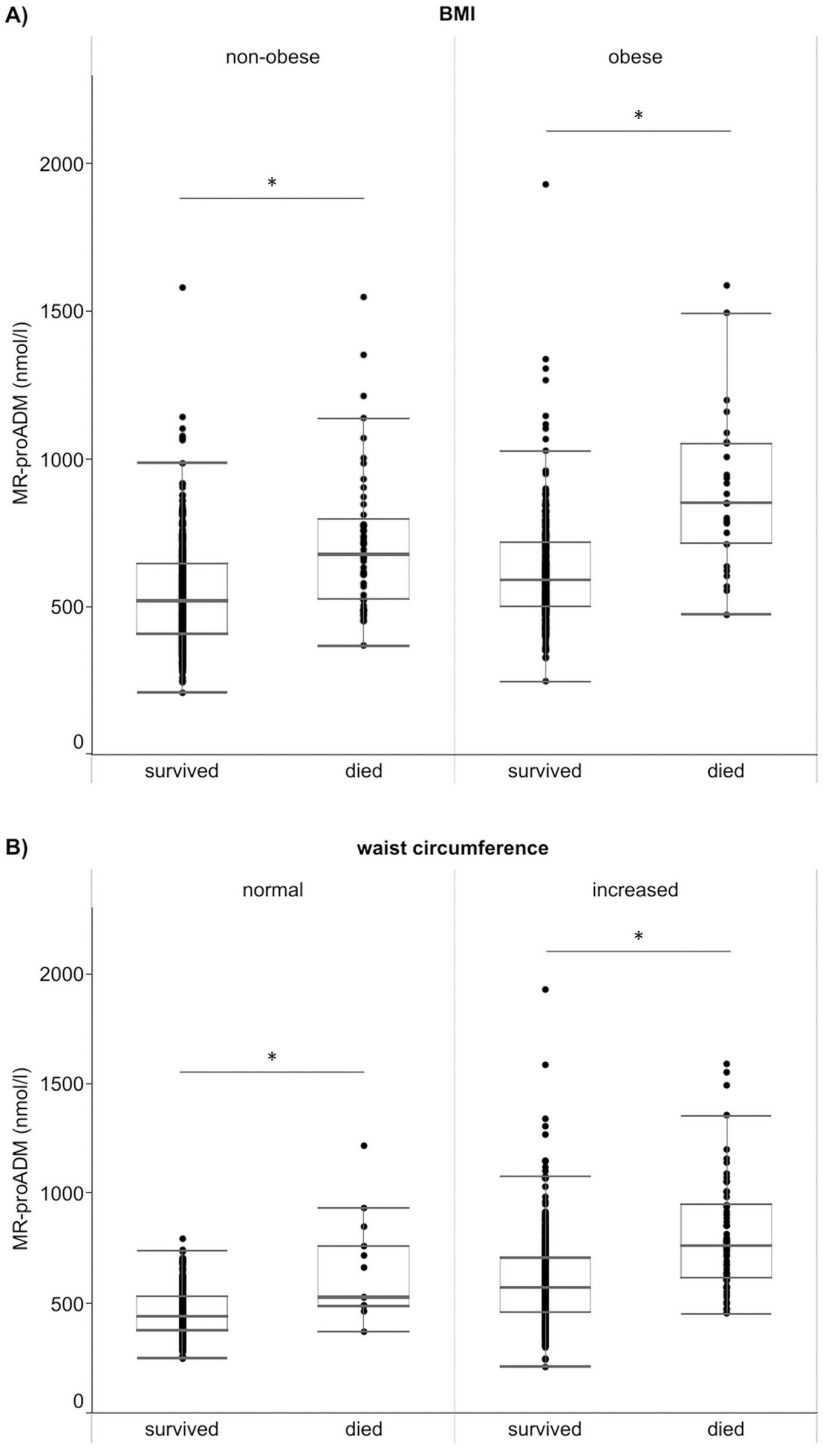
MR-proADM levels stratified by survival status (survived vs. died) in different BMI (A) and waist circumference (B) groups. Non-obese: BMI < 30 kg/m^2^, obese: BMI ≥ 30 kg/m^2^. Increased waist circumference: ≥ 94 cm in men and ≥ 80 cm in women. Group comparison by Mann-Whitney U-test. *p < 0.001 for comparison survival vs. death.

Furthermore, we analyzed the association between MR-proADM and all-cause mortality stratified by overall overweight/obesity (BMI < 30 vs. ≥ 30 kg/m^2^) and subgroups of abdominal obesity (waist circumference < 80 vs. ≥ 80 cm in women; < 94 vs. ≥ 94 cm in men). Irrespective of BMI and waist circumference subgroups, MR-proADM was higher in participants who died compared to survivors (p < 0.001; Fig 1). The association was stronger in obese compared with in non-obese participants (S1 Table). The p for interaction with BMI was 0.026 in the model adjusted for BMI, the p for interaction waist circumference was 0.034 in the model adjusted for waist circumference.

Likewise, MR-proADM independently associated with cardiovascular mortality (HR 4.28 (95% CI: 2.19–8.39), p < 0.001, S2 Table) with a stronger association in obese compared to non-obese participants, although the interaction term was not significant (p interaction BMI: 0.543).

### Association of MR-proADM with adipokines and biomarkers of subclinical inflammation

**Fig 2:**
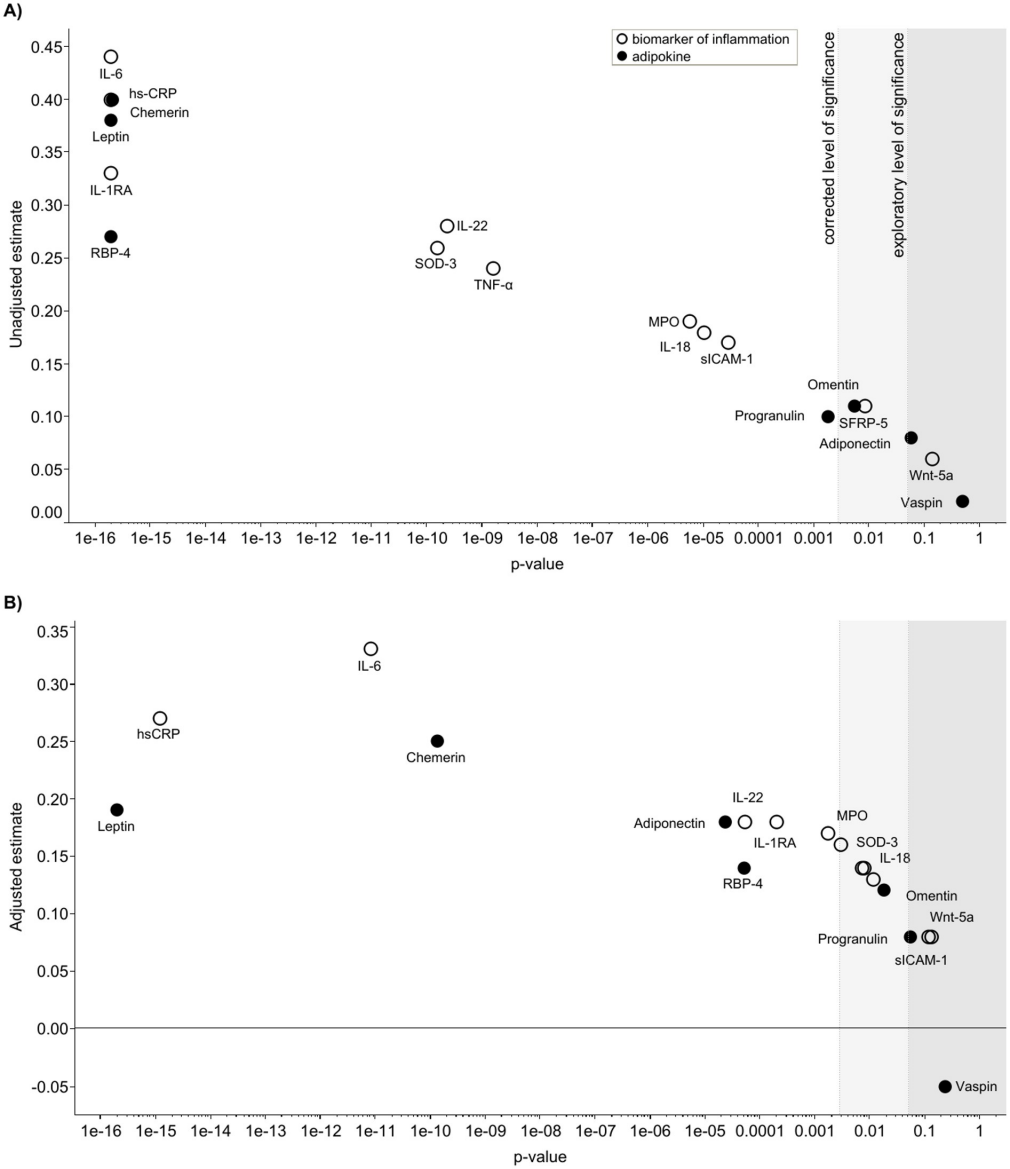
Unadjusted (A) and adjusted (B; adjusted for sex, age, BMI, arterial hypertension, diabetes, eGFR, HDL, smoking, and physical activity) estimates (β coefficient per 1-standard deviation) of the association of MR-proADM with biomarkers of subclinical inflammation (circles) and adipokines (black dots). Exploratory level of significance: p < 0.05 (light grey area); corrected level of significance after Bonferroni correction for multiple testing p < 0.0028 (0.05 ÷ 18); white area).

MR-proADM associated with four out of seven adipokines (leptin, RBP-4, chemerin, and adiponectin) and five out of eleven biomarkers of subclinical inflammation (hsCRP, IL-6, IL-1RA, IL-22, and MPO) after multivariable adjustment (Model 4) and correction for multiple testing using the Bonferroni method (Fig 2 and S3 Table).

There was a trend towards a stronger association of MR-proADM with MPO, IL-22, leptin, and adiponectin in obese compared to non-obese participants (S4 Table). However, after correcting for multiple testing, only leptin (p < 0.001) showed a significant interaction with BMI and waist circumference (S4 Table). MR-proADM was significantly associated with IL-6 in participants with an increased waist circumference (β: 0.39 ± 0.05; p < 0.001), but not in participants with a normal waist circumference (β: 0.06 ± 0.124; p = 0.644) (S4 Table).

### Association of MR-proADM with all-cause mortality in consideration of adipokines and biomarkers of subclinical inflammation

Finally, we examined a possible involvement of the selected adipokines and inflammation markers in the association between MR-proADM and all-cause mortality. Of the biomarkers that significantly associated with MR-proADM, leptin, chemerin, and IL-22 neither associated (p > 0.05) with all-cause mortality in the simple model (model 2; adjusted for sex, age, and BMI) nor in the fully adjusted model (model 4; adjusted for sex, age, BMI, arterial hypertension, diabetes, eGFR, HDL, smoking, and physical activity).

In contrast, RBP-4, hsCRP, adiponectin, IL-6, MPO, and IL-1RA associated with all-cause mortality in both model 2 and 4 (p < 0.05). Hence, we further examined their potential to affect the association of MR-proADM with all-cause mortality. Inclusion of the parameters into the regression models did not substantially attenuate the association of MR-proADM with all-cause mortality (S5 Table). Only the adjustment for IL-6 resulted in an attenuation of the association of MR-proADM with all-cause mortality (HR 1.89 (95% CI: 1.51–2.36)), which persisted in the fully adjusted model (model 4 + IL-6: HR 1.93 (95% CI: 1.41–2.70)). MR-proADM remained significantly associated with all-cause mortality in all models (p < 0.001).

## Discussion

In this study, we observed associations of MR-proADM with all-cause and cardiovascular mortality in the population-based KORA cohort, which were independent of (abdominal) obesity and other “traditional” cardiovascular risk factors, adipokines, and biomarkers of subclinical inflammation. To our knowledge, the association of MR-proADM with a large number of adipokines and biomarkers of subclinical inflammation was analyzed for the first time.

ADM and also MR-proADM have been suggested to be involved in many physiological processes with a possible impact on cardiovascular and metabolic regulation [4]. ADM has direct effects on blood vessels but may also act via central and renal mechanisms. Renal effects may explain the inverse association between MR-proADM and the eGFR, which was also found in the present study. We further confirmed an association between MR-proADM concentrations and cardiovascular risk factors. We observed that MR-proADM levels were dependent on age, which in turn is the major risk factor for mortality. Nevertheless, after adjustment for cardiovascular and metabolic risk factors, MR-proADM was still related to mortality, leaving some of the associations unexplained.

Regarding metabolic regulation, hyperglycemia, obesity, and dyslipidemia are associated with ADM levels [4]. ADM and its receptor have been found to be expressed in adipose tissue and ADM has therefore been claimed to be an adipokine [5, 6]. Additionally, ADM acts on adipose tissue to decrease lipolysis via the inhibition of beta-adrenergic pathways and thereby possibly affects plasma lipid concentrations [5]. In line, we previously demonstrated that increased MR-proADM levels were associated with increased triglyceride and decreased HDL cholesterol concentrations [17]. These metabolic connections may explain the link between increased MR-proADM levels and the metabolic syndrome [17]. However, the association of MR-proADM levels with mortality was independent of metabolic risk factors.

We further examined whether abdominal rather than overall body fat affects the association between MR-proADM and mortality. Our results show a strong positive association between MR-proADM and waist circumference, a surrogate marker for abdominal fat deposition, yet adjustment for waist circumference did not substantially attenuate the association of MR-proADM with all-cause and cardiovascular mortality in our study. However, we found that the association between MR-proADM and mortality was stronger in obese than in non-obese participants. The literature provides some evidence for a link between metabolic characteristics of adipose tissue and (MR-pro)ADM levels. TNF-α was found to increase the amount of ADM mRNA in omental fat, linking (MR-pro)ADM to subclinical inflammation [30]. On the other hand, ADM was suggested to reduce proinflammatory cytokines and counteract the inflammatory effect of cytokines and reactive oxygen species [4, 28, 29]. Hence, it is conceivable that MR-proADM correlates with adverse metabolic derangements, such as an altered adipokine and cytokine profile along with low-grade inflammation of the adipose tissue, which might be more likely to be present in obese compared to non-obese individuals and may partly mediate the association between MR-proADM and increased mortality. In the current study, MR-proADM was associated with several adipokines (leptin, RBP-4, chemerin, and adiponectin) and biomarkers of subclinical inflammation (hsCRP, IL-6, MPO, IL-22, and IL-1RA). some of these associations tended to be stronger in obese than in non-obese participants. we found a strong association between MR-proADM and IL-6 in participants with an increased waist circumference, but not in participants with a normal waist circumference. Preclinical [42–44] and clinical data [45] point towards a modulating effect of ADM on IL-6 under different inflammatory conditions. Furthermore, plasma ADM levels have been found to be related to a single nucleotide polymorphism of the *IL6* gene [46]. In the present study, we observed that addition of IL-6 to the multivariable model attenuated the association between MR-proADM and all-cause mortality, although the association remained significant.

Besides, MR-proADM was associated with leptin and this association was significantly stronger in participants with an increased BMI or waist circumference compared to normal-weight participants, possibly indicating a different activity profile of fat tissue in obese individuals. Both ADM and leptin display vasoprotective propeterties on the one hand, but are also related to cardiovascular risk factors beyond BMI and waist circumference, including arterial hypertension and vascular alterations on the other hand [47, 48]. However, since leptin was not associated with mortality in our study, we did not examine a possible influence of leptin on the association of MR-proADM with mortality.

### Study limitations

The KORA study was conducted in Southern Germany and thus included mainly Western European participants. Consequently, our results might not be applicable to individuals of other ethnicities. Several investigated variables were available only in a subgroup of participants aged ≥ 62 years, limiting the power and generalizability of these results due to the lower number and selected age group. Although we examined a total of 18 adipokines/biomarkers of subclinical inflammation, low-grade adipose tissue inflammation might still have not been represented comprehensively. Similarly, residual confounding by possible unmeasured confounders cannot be excluded. In case of medication with antihypertensive or glucose lowering agents, the diagnoses arterial hypertension and diabetes mellitus were made independently of the measured blood pressure and oral glucose tolerance test, given that the patients were aware of the diagnoses. The observational nature of the study precludes statements about causality. Due to the relatively low number of cardiovascular deaths, subgroup analyses were limited. A major strength of the present study is its population-based design with a high variety of phenotypes and standardized sampling methods that enable high inter-individual comparability of the obtained data.

## Conclusions

In sum, MR-proADM was strongly associated with all-cause and cardiovascular mortality in the population-based KORA F4 study. This association was independent of classical cardiovascular and metabolic risk factors, including BMI. Further, there was no evidence that abdominal obesity (surrogate waist circumference) was a stronger confounding factor than whole body fat (surrogate BMI). From the selected adipokines and biomarkers of subclinical inflammation, only IL-6 attenuated the association of MR-proADM with all-cause mortality. Future studies should examine whether further cytokines or metabolic alterations of subclinical inflammation might be involved in the association between (MR-pro)ADM and mortality, thereby illuminating its underlying pathophysiology.

## Supporting information

S1 Table Hazard ratios (95% confidence interval) of the association between MR-proADM and all-cause mortality (per standard deviation), stratified by BMI and waist circumference. Non-obese: BMI < 30 kg/m^2^, obese: BMI ≥ 30 kg/m^2^. Increased waist circumference: ≥ 94 cm in men and ≥ 80 cm in women. ^**a**^ Model 4: adjusted for sex, age, BMI, arterial hypertension, diabetes, eGFR, HDL cholesterol, smoking, and physical activity; ^**b**^ Model 5: adjusted for sex, age, waist circumference, arterial hypertension, diabetes, eGFR, HDL cholesterol, smoking, and physical activity.
(DOCX)

S2 Table Hazard ratios (95% confidence interval) of the association between MR-proADM and cardiovascular mortality (per standard deviation), stratified by BMI. Non-obese: BMI < 30 kg/m^2^, obese: BMI ≥ 30 kg/m^2^. No analysis of subgroups of waist circumference due to small group sizes. ^**a**^ Model 4: adjusted for sex, age, BMI, arterial hypertension, diabetes, eGFR, HDL cholesterol, smoking and physical activity; ^**b**^ Model 5: adjusted for sex, age, waist circumference, arterial hypertension, diabetes, eGFR, HDL cholesterol, smoking and physical activity.
(DOCX)

S3 Table Beta estimates (β ± standard error) of the association of MR-proADM with adipokines and biomarkers of subclinical inflammation. Bold indicates significance after multivariable adjustment and Bonferroni correction for multiple testing (p < 0.0028 (0.05 ÷ 18)). ^**a**^ Model 4: adjusted for sex, age, BMI, arterial hypertension, diabetes, eGFR, HDL cholesterol, smoking, and physical activity.
(DOCX)

S4 Table Beta estimates (β ± standard error) of the association of MR-proADM with adipokines and biomarkers of subclinical inflammation stratified by BMI and waist circumference. Non-obese: BMI < 30 kg/m^2^, obese: BMI ≥ 30 kg/m^2^. Increased waist circumference: ≥ 94 cm in men and ≥ 80 cm in women. Bold indicates significance after multivariable adjustment and Bonferroni correction for multiple testing for the interaction terms (p < 0.0028 (0.05 ÷ 18)). ^a^ Adjusted for sex, age, BMI, arterial hypertension, diabetes, eGFR, HDL, smoking, and physical activity. ^b^ In the model adjusted for BMI. ^c^ In the model adjusted for waist circumference.
(DOCX)

S5 Table Hazard ratios (95% confidence interval) of the association between MR-proADM and all-cause mortality (per 1-standard deviation). ^a^ Model 4: Adjustment for sex, age, BMI, arterial hypertension, diabetes, eGFR, HDL, smoking and physical activity.
(DOCX)
