# Supplementary material for: Association of circulating MR-proADM with all-cause and cardiovascular mortality in the general population: Results from the KORA F4 cohort study
Source: PLoS One. 2022 Jan 6;17(1):e0262330. doi: 10.1371/journal.pone.0262330 (PMC8735665; doi:10.1371/journal.pone.0262330)
Supplement: S1 Table — Non-obese: BMI < 30 kg/m2, obese: BMI ≥ 30 kg/m2. Increased waist circumference: ≥ 94 cm in men and ≥ 80 cm in women. a Model 4: adjusted for sex, age, BMI, arterial hypertension, diabetes, eGFR, HDL cholesterol, smoking, and physical activity; b Model 5: adjusted for sex, age, waist circumference, arterial hypertension, diabetes, eGFR, HDL cholesterol, smoking, and physical activity. (DOCX) [file pone.0262330.s001.docx]

**S1 Table. Hazard ratios (95% confidence interval) of the association between MR-proADM and all-cause mortality (per standard deviation), stratified by BMI and waist circumference.** Non-obese: BMI < 30 kg/m², obese: BMI ≥ 30 kg/m². Increased waist circumference: ≥ 94 cm in men and ≥ 80 cm in women.

| **subgroups** | **n participants/events** | **HR (95% CI)** | **p-value** | **HR (95% CI)** | **p-value** |
| --- | --- | --- | --- | --- | --- |
|  |  | **Model 4^a^** | | **Model 5^b^** | |
| Total cohort | 1551/138 | 2.37 (1.72-3.26) | < 0.001 | 2.31 (1.67-3.20) | < 0.001 |
| Non-obese | 1160/92 | 1.75 (1.19-2.55) | < 0.001 | 1.69 (1.15-2.49) | 0.009 |
| Obese | 391/46 | 5.91 (3.22-10.83) | < 0.001 | 5.32 (2.96-9.57) | < 0.001 |
| Normal waist circumference | 505/23 | 1.83 (0.72-4.68) | 0.202 | 1.89 (0.73-4.93) | 0.190 |
| Increased waist circumference | 1046/115 | 2.51 (1.77-3.56) | < 0.001 | 2.47 (1.74-3.52) | < 0.001 |

**^a^** Model 4: adjusted for sex, age, BMI, arterial hypertension, diabetes, eGFR, HDL cholesterol, smoking, and physical activity;

**^b^** Model 5: adjusted for sex, age, waist circumference, arterial hypertension, diabetes, eGFR, HDL cholesterol, smoking, and physical activity.
