# Supplementary material for: Association of circulating MR-proADM with all-cause and cardiovascular mortality in the general population: Results from the KORA F4 cohort study
Source: PLoS One. 2022 Jan 6;17(1):e0262330. doi: 10.1371/journal.pone.0262330 (PMC8735665; doi:10.1371/journal.pone.0262330)
Supplement: S2 Table — Non-obese: BMI < 30 kg/m2, obese: BMI ≥ 30 kg/m2. No analysis of subgroups of waist circumference due to small group sizes. a Model 4: adjusted for sex, age, BMI, arterial hypertension, diabetes, eGFR, HDL cholesterol, smoking and physical activity; b Model 5: adjusted for sex, age, waist circumference, arterial hypertension, diabetes, eGFR, HDL cholesterol, smoking and physical activity. (DOCX) [file pone.0262330.s002.docx]

**S2 Table. Hazard ratios (95% confidence interval) of the association between MR-proADM and cardiovascular mortality (per standard deviation), stratified by BMI.** Non-obese: BMI < 30 kg/m², obese: BMI ≥ 30 kg/m². No analysis of subgroups of waist circumference due to small group sizes.

| **Participants (n/number of events)** | **HR (95% CI)** | **p-value** | **HR (95% CI)** | **p-value** |
| --- | --- | --- | --- | --- |
|  | **Model 4^a^** | | **Model 5^b^** | |
| Total cohort (1551/36) | 4.28 (2.19-8.39) | < 0.001 | 4.44 (2.25-8.76) | < 0.001 |
| Non-obese (1160/27) | 2.77 (1.23-5.71) | 0.006 | 2.74 (1.30-5.78) | 0.008 |
| Obese (391/9) | 43.71 (5.40-353.8) | < 0.001 | 24.97 (4.26-146.22) | < 0.001 |

**^a^** Model 4: adjusted for sex, age, BMI, arterial hypertension, diabetes, eGFR, HDL cholesterol, smoking and physical activity;

**^b^** Model 5: adjusted for sex, age, waist circumference, arterial hypertension, diabetes, eGFR, HDL cholesterol, smoking and physical activity.
