# Supplementary material for: Association of circulating MR-proADM with all-cause and cardiovascular mortality in the general population: Results from the KORA F4 cohort study
Source: PLoS One. 2022 Jan 6;17(1):e0262330. doi: 10.1371/journal.pone.0262330 (PMC8735665; doi:10.1371/journal.pone.0262330)
Supplement: S3 Table — Bold indicates significance after multivariable adjustment and Bonferroni correction for multiple testing (p < 0.0028 (0.05 ÷ 18)). a Model 4: adjusted for sex, age, BMI, arterial hypertension, diabetes, eGFR, HDL cholesterol, smoking, and physical activity. (DOCX) [file pone.0262330.s003.docx]

**S3 Table. Beta estimates (β ± standard error) of the association of MR-proADM with adipokines and biomarkers of subclinical inflammation.** Bold indicates significance after multivariable adjustment and Bonferroni correction for multiple testing (p < 0.0028 (0.05 ÷ 18)).

|  | **n** | **β coefficient ± SE** | **p-value** | **β coefficient ± SE** | **p-value** |
| --- | --- | --- | --- | --- | --- |
| **Adipokines** |  | **Without adjustment** | | **Model 4^a^** | |
| Leptin | 1549 | 0.38 ± 0.02 | < 0.001 | 0.19 ± 0.02 | **< 0.001** |
| RBP-4 | 1549 | 0.27 ± 0.02 | < 0.001 | 0.14 ± 0.03 | **< 0.001** |
| Chemerin | 1055 | 0.40 ± 0.03 | < 0.001 | 0.25 ± 0.04 | **< 0.001** |
| Progranulin | 1055 | 0.10 ± 0.03 | 0.002 | 0.09 ± 0.04 | 0.039 |
| Vaspin | 1055 | 0.02 ± 0.03 | 0.482 | -0.05 ± 0.04 | 0.237 |
| Adiponectin | 606 | 0.08 ± 0.04 | 0.057 | 0.19 ± 0.04 | **< 0.001** |
| Omentin | 606 | 0.11 ± 0.04 | 0.006 | 0.12 ± 0.05 | 0.018 |
| **Biomarkers of subclinical inflammation** | | **Without adjustment** | | **Model 4^a^** | |
| hsCRP | 1551 | 0.40 ± 0.02 | < 0.001 | 0.27 ± 0.03 | **< 0.001** |
| IL-6 | 603 | 0.44 ± 0.04 | < 0.001 | 0.32 ± 0.05 | **< 0.001** |
| TNF-α | 603 | 0.24 ± 0.04 | < 0.001 | 0.14 ± 0.05 | 0.009 |
| IL-18 | 603 | 0.18 ± 0.04 | < 0.001 | 0.14 ± 0.05 | 0.007 |
| SFRP-5 | 606 | 0.11 ± 0.04 | 0.008 | 0.13 ± 0.05 | 0.012 |
| Wnt-5a | 606 | 0.06 ± 0.04 | 0.139 | 0.09 ± 0.05 | 0.131 |
| sICAM-1 | 603 | 0.17 ± 0.04 | < 0.001 | 0.08 ± 0.05 | 0.142 |
| IL-1RA | 603 | 0.33 ± 0.04 | < 0.001 | 0.18 ± 0.05 | **< 0.001** |
| IL-22 | 603 | 0.28 ± 0.04 | < 0.001 | 0.19 ± 0.04 | **< 0.001** |
| MPO | 603 | 0.19 ± 0.04 | < 0.001 | 0.17 ± 0.05 | **0.002** |
| SOD-3 | 603 | 0.26 ± 0.04 | < 0.001 | 0.16 ± 0.05 | 0.003 |

**^a^** Model 4: adjusted for sex, age, BMI, arterial hypertension, diabetes, eGFR, HDL cholesterol, smoking, and physical activity
