# Supplementary material for: Association of circulating MR-proADM with all-cause and cardiovascular mortality in the general population: Results from the KORA F4 cohort study
Source: PLoS One. 2022 Jan 6;17(1):e0262330. doi: 10.1371/journal.pone.0262330 (PMC8735665; doi:10.1371/journal.pone.0262330)
Supplement: S4 Table — Non-obese: BMI < 30 kg/m2, obese: BMI ≥ 30 kg/m2. Increased waist circumference: ≥ 94 cm in men and ≥ 80 cm in women. Bold indicates significance after multivariable adjustment and Bonferroni correction for multiple testing for the interaction terms (p < 0.0028 (0.05 ÷ 18)). a Adjusted for sex, age, BMI, arterial hypertension, diabetes, eGFR, HDL, smoking, and physical activity. b In the model adjusted for BMI. c In the model adjusted for waist circumference. (DOCX) [file pone.0262330.s004.docx]

**S4 Table. Beta estimates (β ± standard error) of the association of MR-proADM with adipokines and biomarkers of subclinical inflammation stratified by BMI and waist circumference.** Non-obese: BMI < 30 kg/m², obese: BMI ≥ 30 kg/m². Increased waist circumference: ≥ 94 cm in men and ≥ 80 cm in women. Bold indicates significance after multivariable adjustment and Bonferroni correction for multiple testing for the interaction terms (p < 0.0028 (0.05 ÷ 18)).

|  |  | **BMI** | | | | | **waist circumference** | | | | |
| --- | --- | --- | --- | --- | --- | --- | --- | --- | --- | --- | --- |
|  |  | **subgroup** | **n** | **β coefficient ± SE ^a^** | **p-value ^a^** | **p interaction ^b^** | **subgroup** | **n** | **β coefficient ± SE ^a^** | **p-value ^a^** | **p interaction ^c^** |
| **Adipokines** | | | | | | | | | | | |
| Leptin |  | Non-obese | 1158 | 0.18 ± 0.03 | < 0.001 | **< 0.001** | Normal | 505 | 0.14 ± 0.04 | < 0.001 | **< 0.001** |
|  |  | Obese | 391 | 0.26 ± 0.05 | < 0.001 |  | increased | 1044 | 0.21 ± 0.03 | < 0.001 |  |
| RBP-4 |  | Non-obese | 1158 | 0.15 ± 0.04 | < 0.001 | 0.038 | Normal | 505 | 0.13 ± 0.06 | 0.030 | 0.466 |
|  |  | Obese | 391 | 0.12 ± 0.08 | 0.142 |  | increased | 1044 | 0.14 ± 0.04 | 0.001 |  |
| Chemerin |  | Non-obese | 757 | 0.24 ± 0.04 | < 0.001 | 0.374 | Normal | 284 | 0.36 ± 0.07 | < 0.001 | 0.803 |
|  |  | Obese | 298 | 0.23 ± 0.08 | 0.004 |  | increased | 771 | 0.20 ± 0.05 | < 0.001 |  |
| Adiponectin |  | Non-obese | 423 | 0.16 ± 0.05 | 0.001 | 0.072 | Normal | 105 | 0.22 ± 0.10 | 0.038 | 0.715 |
|  |  | Obese | 183 | 0.21 ± 0.09 | 0.016 |  | increased | 501 | 0.19 ± 0.05 | < 0.001 |  |
| **Markers of subclinical inflammation** | | | | | | | | | | | |
| hsCRP |  | Non-obese | 1160 | 0.28 ± 0.04 | < 0.001 | 0.455 | Normal | 505 | 0.23 ± 0.07 | 0.003 | 0.797 |
|  |  | Obese | 391 | 0.24 ± 0.06 | < 0.001 |  | increased | 1046 | 0.28 ± 0.04 | < 0.001 |  |
| IL-6 |  | Non-obese | 421 | 0.34 ± 0.06 | < 0.001 | 0.335 | Normal | 104 | 0.06 ± 0.13 | 0.646 | 0.478 |
|  |  | Obese | 182 | 0.28 ± 0.08 | < 0.001 |  | increased | 499 | 0.38 ± 0.05 | < 0.001 |  |
| IL-22 |  | Non-obese | 421 | 0.14 ± 0.05 | 0.007 | 0.289 | Normal | 104 | 0.13 ± 0.10 | 0.223 | 0.823 |
|  |  | Obese | 182 | 0.31 ± 0.09 | < 0.001 |  | increased | 499 | 0.21 ± 0.05 | < 0.001 |  |
| IL-1RA |  | Non-obese | 421 | 0.20 ± 0.06 | < 0.001 | 0.706 | Normal | 104 | 0.19 ± 0.12 | 0.113 | 0.918 |
|  |  | Obese | 182 | 0.15 ± 0.09 | 0.116 |  | increased | 499 | 0.19 ± 0.05 | < 0.001 |  |
| MPO |  | Non-obese | 421 | 0.09 ± 0.06 | 0.180 | 0.102 | Normal | 104 | -0.20 ± 0.12 | 0.114 | 0.023 |
|  |  | Obese | 182 | 0.32 ± 0.09 | 0.001 |  | increased | 499 | 0.24 ± 0.06 | < 0.001 |  |

^a^ Adjusted for sex, age, BMI, arterial hypertension, diabetes, eGFR, HDL, smoking, and physical activity.

^b^ In the model adjusted for BMI

^c^ In the model adjusted for waist circumference
