# Supplementary material for: Association of circulating MR-proADM with all-cause and cardiovascular mortality in the general population: Results from the KORA F4 cohort study
Source: PLoS One. 2022 Jan 6;17(1):e0262330. doi: 10.1371/journal.pone.0262330 (PMC8735665; doi:10.1371/journal.pone.0262330)
Supplement: S5 Table — a Model 4: Adjustment for sex, age, BMI, arterial hypertension, diabetes, eGFR, HDL, smoking and physical activity. (DOCX) [file pone.0262330.s005.docx]

**S5 Table. Hazard ratios (95% confidence interval) of the association between MR-proADM and all-cause mortality (per 1-standard deviation).**

| **Adjustments** | **N participants/events** | **HR (95% CI)** | **p-value** |
| --- | --- | --- | --- |
| **Total cohort** |  |  |  |
| Without adjustment | 1549/138 | 3.23 (2.71-3.83) | < 0.001 |
| Model 4^a^ | 1549/138 | 2.35 (1.71-3.22) | < 0.001 |
| RBP-4 | 1549/138 | 3.69 (3.04-4.50) | < 0.001 |
| Model 4^a^ + RBP-4 | 1549/138 | 2.55 (1.85-3.52) | < 0.001 |
| hsCRP | 1551/138 | 3.05 (2.52-3.68) | < 0.001 |
| Model 4^a^ + hsCRP | 1551/138 | 2.14 (1.55-2.97) | < 0.001 |
| **Participants aged ≥ 62 years** |  |  |  |
| Without adjustment | 606/107 | 2.24 (1.84-2.73) | < 0.001 |
| Model 4^a^ | 606/107 | 2.25 (1.66-3.09) | < 0.001 |
| Adiponectin | 606/107 | 2.24 (1.83-2.73) | < 0.001 |
| Model 4^a^ + adiponectin | 606/107 | 2.01 (1.48-2.74) | < 0.001 |
| Model 4^a^ | 603/106 | 2.27 (1.65-3.12) | < 0.001 |
| IL-6 | 603/106 | 1.89 (1.51-2.36) | < 0.001 |
| Model 4^a^ + IL-6 | 603/106 | 1.93 (1.41-2.70) | < 0.001 |
| MPO | 603/106 | 2.13 (1.74-2.60) | < 0.001 |
| Model 4^a^ + MPO | 603/106 | 2.16 (1.57-2.98) | < 0.001 |
| IL-1RA | 603/106 | 2.19 (1.59-3.02) | < 0.001 |
| Model 4^a^ + IL-1RA | 603/106 | 2.13 (1.55-2.92) | < 0.001 |

^a^ Model 4: Adjustment for sex, age, BMI, arterial hypertension, diabetes, eGFR, HDL, smoking and physical activity.
